# Chitosan nanocomposites based on distinct inorganic fillers for biomedical applications

**DOI:** 10.1080/14686996.2016.1229104

**Published:** 2016-10-10

**Authors:** Duarte Moura, João F. Mano, Maria C. Paiva, Natália M. Alves

**Affiliations:** ^a^ 3B’s Research Group, Biomaterials, Biodegradables and Biomimetics, University of Minho, Headquarters of the European Institute of Excellence on Tissue Engineering and Regenerative Medicine, Guimarães, Portugal; ^b^ ICVS/3B’s, Associate PT Government Laboratory, Braga, Guimarães, Portugal; ^c^ Institute for Polymers and Composites/I3 N, Department of Polymer Engineering, University of Minho, Guimarães, Portugal

**Keywords:** Chitosan, polymer nanocomposites, biomedical applications, 20 Organic and soft materials (colloids, liquid crystals, gel, polymers), 101 Self-assembly / Self-organized materials, 102 Porous / Nanoporous / Nanostructured materials, 103 Composites, 211 Scaffold / Tissue engineering / Drug delivery, 212 Surface and interfaces, 306 Thin film / Coatings, 104 Carbon and related materials

## Abstract

Chitosan (CHI), a biocompatible and biodegradable polysaccharide with the ability to provide a non-protein matrix for tissue growth, is considered to be an ideal material in the biomedical field. However, the lack of good mechanical properties limits its applications. In order to overcome this drawback, CHI has been combined with different polymers and fillers, leading to a variety of chitosan-based nanocomposites. The extensive research on CHI nanocomposites as well as their main biomedical applications are reviewed in this paper. An overview of the different fillers and assembly techniques available to produce CHI nanocomposites is presented. Finally, the properties of such nanocomposites are discussed with particular focus on bone regeneration, drug delivery, wound healing and biosensing applications.

## Graphical abstract

**Figure:**
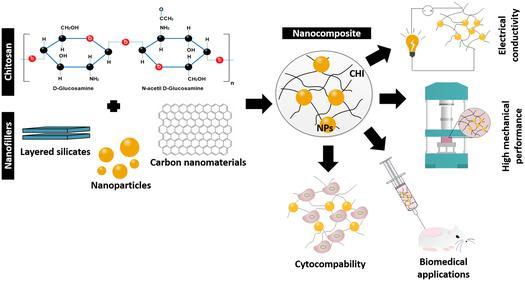

## 1. Introduction

The increasing need to develop green polymeric materials with improved thermal stability, gas barrier properties, strength and biodegradation, has led to the development of composite materials based on natural polymers.[1]

Chitosan (CHI) is a linear semicrystalline polysaccharide, obtained by deacetylation of chitin and composed by N-acetyl D-glucosamine and D-glucosamine units, linked through β (1→4) glycosidic bonds.[2,3] Chitin or poly(β-(1→4)-N-acetyl-D-glucosamine) is synthetized by living organisms, being the structural component of the shells of crustaceous, cell walls of fungi and exoskeletons of insects.[3–5] Typically, shrimp and crab shell waste are the primary source for isolation and purification of this polysaccharide.[3,6–8] When subjected to a deacetylation process, under alkaline conditions, part of the N-acetyl groups are lost and when at least 60% of the chitin units are D-glucosamine this polysaccharide is named CHI[9,10] A soluble CHI is obtained after deacetylation, requiring 80–85% of free amino groups and acidic pH.[5] The free amino groups confer exceptional chemical and physical properties, among which are worth mentioning the positive charge in aqueous solutions at low pH.[5] At pH values above its pKa ≈ 6.5, the polymer loses the positive charge and precipitates, which makes CHI a pH responsive material.[5]

Moreover, CHI exhibits outstanding properties, such as biocompatibility and biodegradability,[11] ability to be sterilized by any method without losing its properties,[12] along with antibacterial, antifungal, mucoadhesive, analgesic and hemostatic properties.[4,13–16] Besides, CHI has been shown to provide a non-protein matrix for tissue growth with potential to stimulate cell proliferation and tissue organization.[13] Its chemical structure can be modified in order to improve the mentioned features, and also its solubility.[2,13] Usually, these modifications are achieved by copolymerization, grafting the free amino groups or the $C_{3}$ and *C*_6_ carbons in the CHI units with different types of moieties, including alkyl and carboxymethyl groups, resulting in different CHI derivatives.[17,18] However, CHI still exhibits some limitations such as the lack of an appropriate biological response, partly caused by the weak mechanical properties for many applications (e.g. low stiffness) and high water content.[19] To overcome these problems, CHI composites with polymers or fillers have been produced.[5] The use of nanofillers is particularly interesting due to their high surface area, providing a large interface that, combined with high interfacial strength, will increase the load transfer capacity and greatly improve the mechanical properties of the composite, eliminating the catastrophic failure caused by poor loading transfer.[20,21]

**Figure 1.**
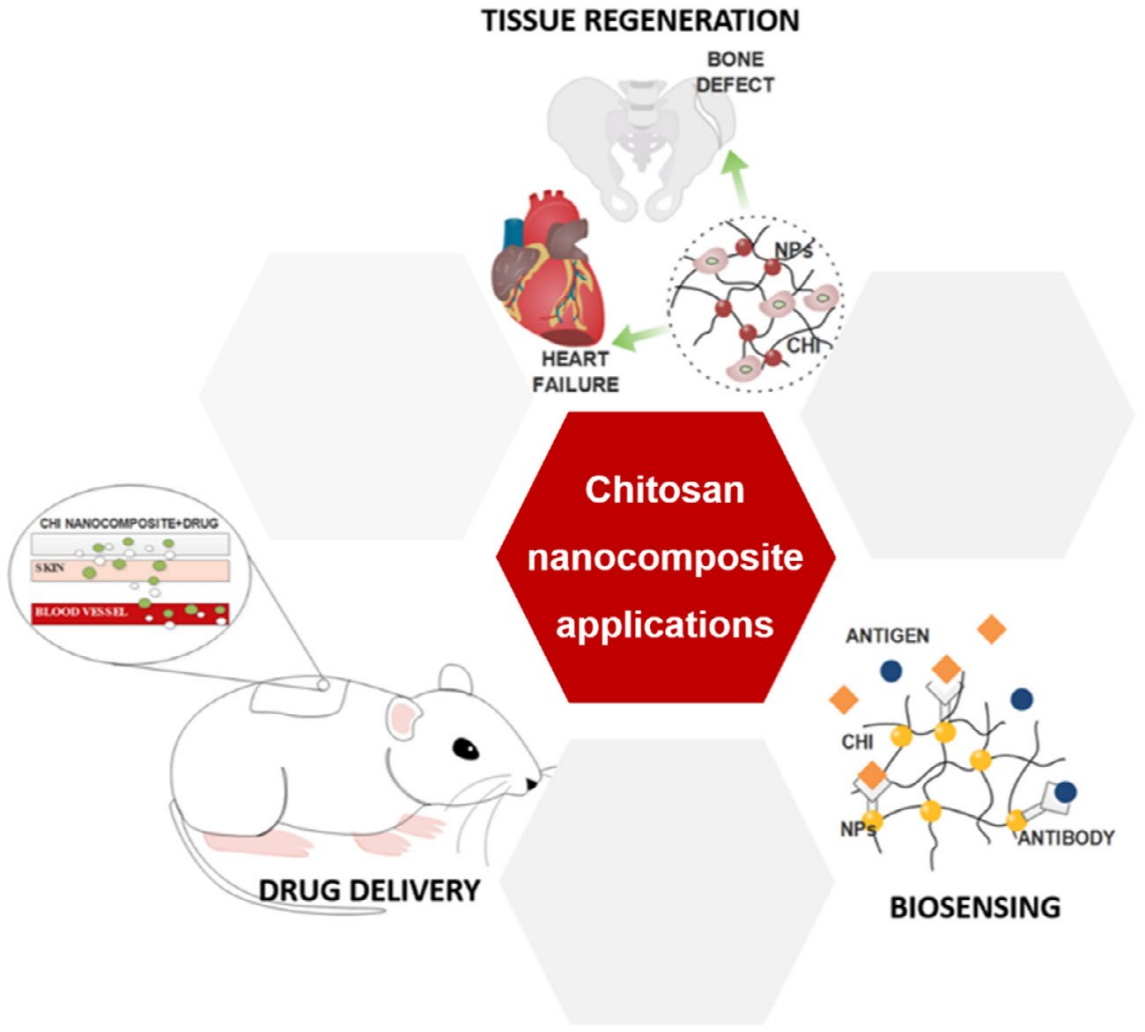
Main biomedical applications of chitosan nanocomposites.

The interesting characteristics of CHI, allied with its ability to be processed in different architectures and combined with different nanomaterials, lead to a wide number of applications, including in the biomedical field, such as drug delivery, tissue regeneration and biosensing [13,22]; see Figure 1.

Nanocomposites based on CHI are able to improve both structural and functional properties of this polysaccharide. In this review, an overview of the main types of CHI nanocomposite materials, their properties and processing strategies will be given. The biomedical applications of these nanocomposites will be discussed.

## 2. Chitosan nanocomposites

Polymer nanocomposites are commonly defined as multiphase materials, where one of the phases exhibit at least one dimension smaller than 100 nm.[23] Typically, two essential key aspects are responsible for the successful development of nanocomposites:

(1) high specific interfacial area; and
(2) controlled stress transfer across the interface.

The first key factor is of major importance concerning the properties of nanocomposites. The large specific area of the nanoparticles provides a high surface-to-volume ratio, resulting in extensive binding between the polymer and the nanofiller. Achieving a strong interfacial bonding is then necessary in order to transfer the excellent properties of the nanoparticles into the composite.[24] Thus, a strong interfacial bonding will allow efficient stress transfer across the interface. This key aspect relates to the bonding energy at the interface, which may be improved through covalent bonding, electrostatic interactions, hydrogen bonding and van der Waals interactions.[25] In order to achieve the desired enhanced mechanical, electrical and optical properties, it is also important that a good nanofiller distribution within the matrix is achieved, overcoming the aggregation tendency that is typical of nanoparticles and which results in decreased interfacial area and poor mechanical properties.[23,24] The interfacial strength plays a major role in the nanocomposites’ ultimate mechanical properties, including toughness, tensile strength and elastic modulus.[26]

Among the different nanofillers used as reinforcement phase, the most used in the development of CHI nanocomposites are: (i) layered silicates, such as clay [27–29]; (ii) metal/ceramic nanoparticles [30–32]; (iii) carbon nanotubes [33–35]; and more recently (iv) graphene based materials.[36–38] In the next subsections, each of these fillers will be analyzed.

### 2.1. Layered silicates

Polymer–clay nanocomposites have been widely studied due to the improved material properties when compared to micro and macro composites.[39] Clays are hydrous silicates or aluminosilicates with high aspect ratio and high intercalation chemistry. Typically, this nanofiller shows a layered structure which contains silicon, aluminum or magnesium, oxygen and hydroxyl with various associated cations.[40]

**Figure 2.**
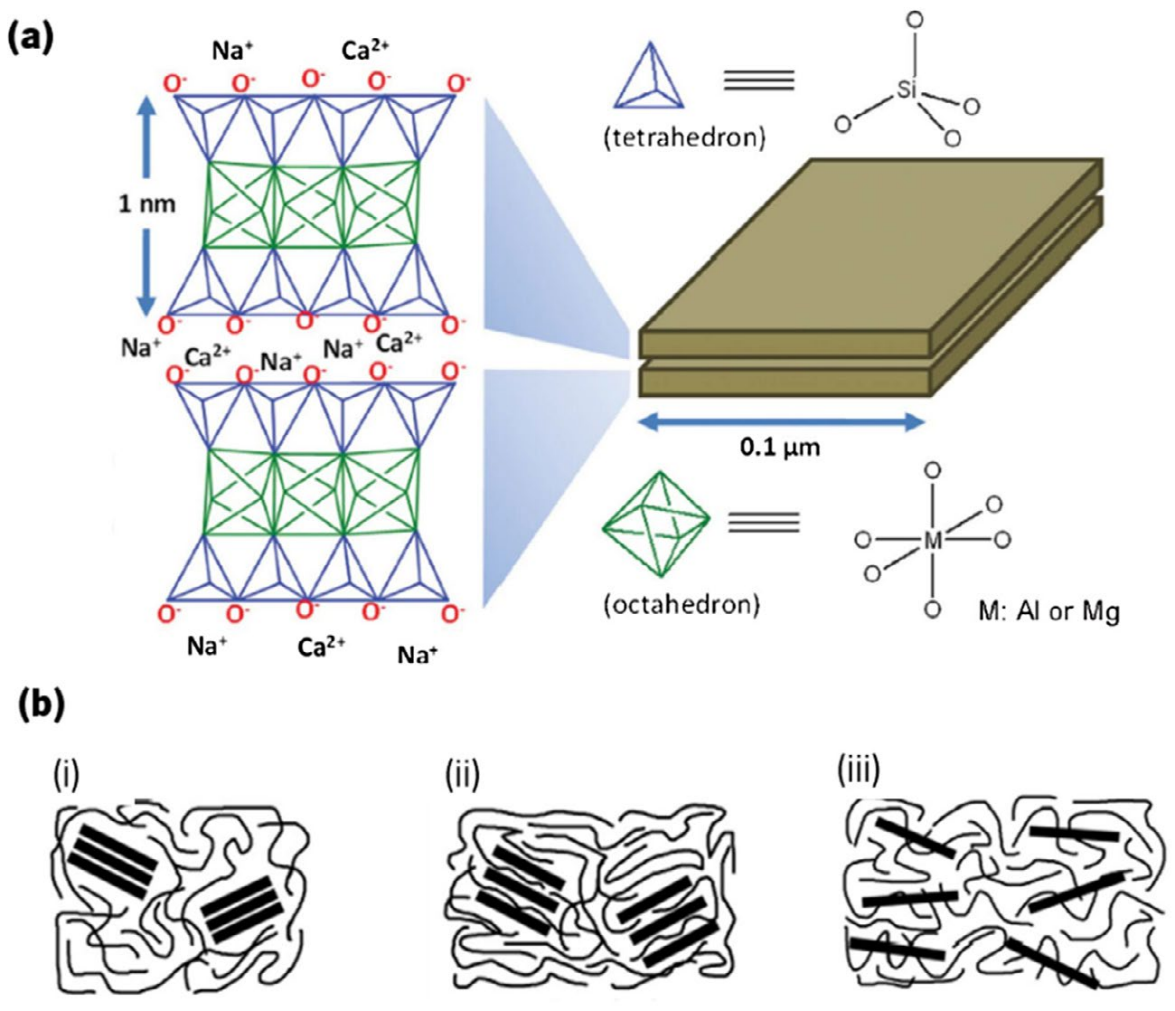
Representation of the basic units (a) Si-O tetrahedron and Al-O or Al-O octahedron present on the clay minerals [43]. (b) Representation of the different structures resulted from different clay dispersion in the polymeric matrix. (i) Tactoid structures, (ii) intercalated structures and (iii) exfoliated structures [39].

In a more detailed analysis, the layers are built from tetrahedrally bonded silicon (Si) atoms surrounded by four oxygen atoms, and from octahedrally bonded aluminum (Al) or magnesium (Mg) surrounded by eight oxygen atoms (Figure 2(a)).[41] The structure and composition of clays can be classified according to the ratio of silica and alumina sheets: (i) kaolins (1:1 type), which consist in one tetrahedral sheet and one octahedral sheet linked by hydrogen bonding; (ii) smectites (2:1 type), which result from the presence of one octahedral sheet between two tetrahedral sheets – in this conformation, the aluminum ions present in the octahedron sheets may be substituted by ${\text{Fe}}^{2+}$, ${\text{Mg}}^{2+}$ or ${\text{Li}}^{+}$, and the ${\text{Si}}^{4+},$ present in tetrahedral sheet by ${\text{Al}}^{3+}$, resulting in an overall negative charge; and (iii) layered silicate acids, where silicon tetrahedron sheets with different thickness are interlayered with alkali metal cations.[40,42,43]

Clays are hydrophilic in nature and need to be modified for mixing with organic polymers, usually by treatment with ammonium or phosphonium ions, in order to increase the interlayer space between sheets and facilitate the diffusion of the polymer.[43–45]

For the successful development of nanocomposites with layered silicates, it is important to ensure that the silicate structures are homogeneously dispersed into the polymeric matrix. Commonly, three different dispersion conformations can be obtained: (i) tactoid structures, when the interlayer space of the clay does not expand, not allowing the intercalation of the polymer, which is not desirable [39]; (ii) intercalated structures, when the layered clay structure is maintained but the interlayer distance increases, allowing penetration of the polymer; and (iii) exfoliated structures, when the clay layers are well separated from each other, resulting in a good dispersion and mixing with the polymer phase (Figure 2(b)).[46]

Montmorillonite (MMT) is an aluminosilicate belonging to the smectites group, and is one of the most studied for nanocomposite applications.[47,48] This layered silicate presents a negative charge of 0.67 per unit cell, which attributes to this material a weak acid behavior, and provides a strong capacity for hydration, swelling and dispersion. These characteristics provide MMT affinity to cations, and allow exfoliation of its layers, bonded by weak electrostatic and van der Waals interactions.[43,48]

Since the intercalation of MMT with CHI is feasible due to the electrostatic and hydrogen bond interaction established between the positive amino groups in CHI and the negative sites in MMT, and between amino groups and hydroxyl groups, respectively, these nanocomposites have been the focus of many studies.[27–29,49–51]

### 2.2. Metal/ceramic nanoparticles

Nanoparticles (NPs) combined with polymer matrices lead to a whole new family of materials where the nanocomposites are expected to exhibit improved performances.[52] In fact, the incorporation of inorganic particles into the CHI matrix is performed to achieve improved mechanical properties and, in some cases, to provide bioactivity to an inert material.[53] Usually three main groups of particles are identified as fillers for CHI nanocomposites: bioactive glass, bioactive ceramic nanoparticles and metal nanoparticles.

Bioactive glass (BG) represents a group of surface reactive materials with the ability to bond to physiological structures, such as bone. BG are typically obtained by melt or sol-gel methods.[54] This type of NP is essentially made of silicate with a variable composition of sodium, calcium and phosphorus. Due to their nanometric dimensions, these particles present a high specific surface area with the ability to release ions and to provide a good substrate for protein adsorption.[52,55] Sol-gel methodologies allow the production of nano-sized bioactive particles with controlled compositions.[56–58]

Similarly to the previous fillers, ceramic NPs are also used to form nanocomposites, providing enhanced mechanical properties and improving the interaction with surrounding tissues.[55] In particular, hydroxyapatite nanoparticles (HA NPs) have shown potential for nanocomposite fabrication, showing good osteoconductivity, osteoinductivity, biodegradability and high mechanical strength.[59] However, the brittle character of these materials is detrimental for the composite properties when the ceramic nanoparticles are incorporated at high concentrations.[60]

In the case of metal NPs, these are combined with polymers to provide antibacterial [61] and biosensing [62] characteristics. In fact, nearly all metals were already reported to be used for nanoparticle production, including silver,[62,63] gold,[62,64] and zinc oxide [65] nanoparticles, among others.[66–68]

Independently of the NP type, several parameters may be controlled in order to tune the composites’ properties, including particle size, chemical composition, crystallinity and shape.[69]

### 2.3. Carbon nanotubes (CNTs)

Carbon nanotubes (CNTs) were first identified by Iijima in 1991,[70] and may be described as hexagonal ${\text{sp}}^{2}$ carbon structures rolled up in a tube.[71] These seamless cylinders may present open or closed ends, the latter being capped by hemi-fullerene structures.[72] Two types of CNTs may be identified: (i) single-walled carbon nanotubes (SWNTs); and (ii) multi-walled carbon nanotubes (MWNTs).[73] The first type consists of a single graphene sheet rolled up in a cylinder, presenting a diameter of approximately 1 nm and a length that may reach the range of centimeters, while MWNTs are composed by multiple concentric cylindrical sheets of graphene, held together by van der Waals forces, with an interlayer space of approximately 0.35 nm.[74] The diameter of the MWNTs may range from 5 to 20 nm depending on the number of layers, although it may reach 100 nm.[73,75,76]

Typically, CNTs exhibit a high elastic modulus, reaching 1 TPa (the elastic modulus of diamond is 1.2 TPa), a tensile strength of 63 GPa,[77] and a density of 1.74 g cm^–3^. Under ideal conditions, in the absence of defects of the carbon lattice, CNTs are able to achieve a ballistic electron transport,[78] due to the absence of scattering which reduces the heat loss and allows large current densities to be carried.[79] A room temperature thermal conductivity up to 6000 W m^–1^ K^–1^ has been reported, which is twice as high as that of diamond.[78,80–82]

These remarkable properties may vary according to several factors, such as the present of defects in the atomic arrangement, the diameter and length of the CNTs, the synthetic process used, and impurities present in the structure.[72] One of the most influential features of SWNT properties is their atomic arrangement relative to the nanotube axis. This is described in terms of the tube chirality, defined by the chiral angle. The SWNTs may present different conformations, namely the zig-zag (chiral angle = 0°) and the armchair conformation (chiral angle = 30°). Depending on the conformation, the carbon nanotubes may be classified as metallic, semi-metallic or semi-conducting.[73,83–86]

The excellent properties of CNTs make them attractive for nanocomposite applications. However, for biomedical applications the elimination of metallic impurities is often required, and CNT dispersion in the polymer matrix should also be achieved in order to attain good composite properties.[87] New fabrication methods have been studied to achieve composites with well dispersed CNTs, such as the use of ultrasounds in solution blending.[72] Although the addition of carbon based materials to polymeric matrix resulted in enhanced polymeric properties, in fact, the need to improve the interaction polymer/carbon material to truly take advantage of the carbon materials’ properties should be further explored by induction of defects, and covalent and non-covalent functionalization of CNTs.[88]

### 2.4. Graphene based materials

Graphene consists of a layer of ${\text{sp}}^{2}$ hybridized carbon atoms forming a hexagonal structure, and it is considered the building block of all ${\text{sp}}^{2}$ hybridized carbon allotropes.[26] This carbon material presents a honeycomb-like structure, with a bond length between the carbon atoms of 0.142 nm [89] and it is known as the thinnest 2D material, with a van der Waals thickness of 0.34 nm.[24] Graphene has outstanding properties such as high elastic modulus (≈1 TPa), the highest known intrinsic electrical conductivity of 6 × 10^5^ S m^-1^ and a high thermal conductivity of 5.1 × 10^3^ W m K.[90–92] Moreover it shows a large theoretical specific area of 2630 m^2^ g^-1^, an optical transmittance of 97.7% and a high flexibility.[93,94]

**Figure 3.**
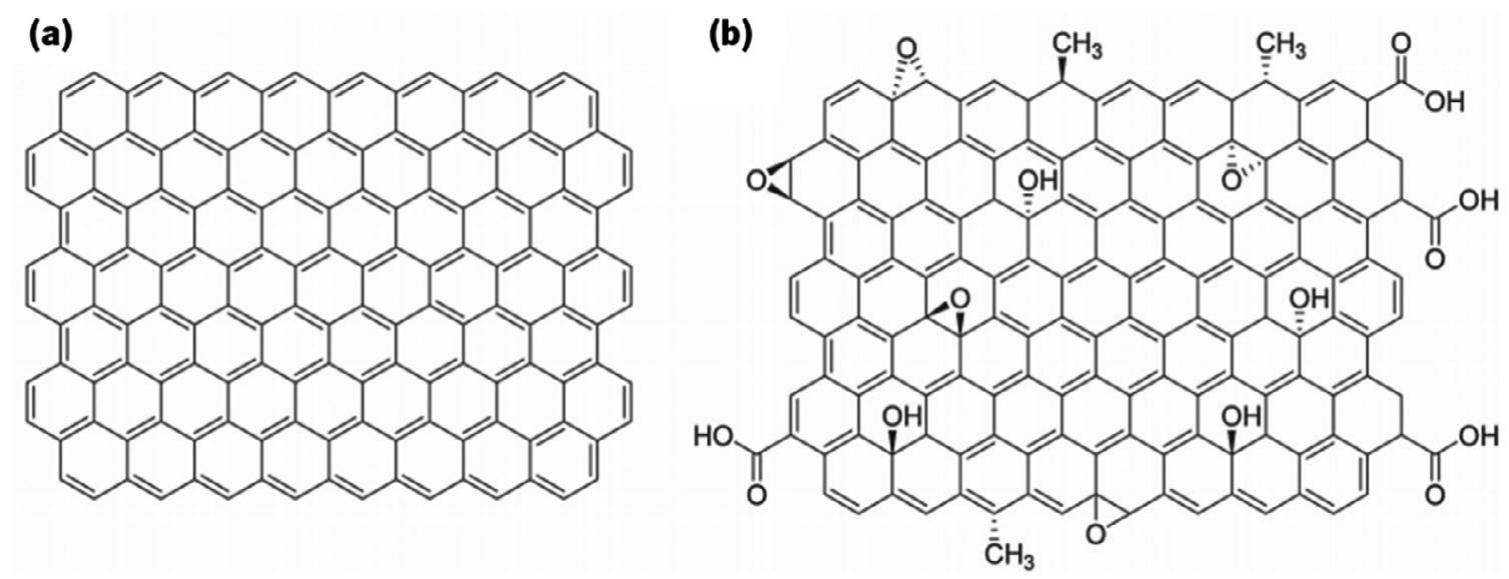
Schematic representation of (a) graphene and (b) graphene oxide sheet [86].

Due to its intrinsic properties and to the possibility of achieving thermally and electrically conductive nanocomposites, graphene has aroused interest from the scientific community.[95–97] Its interfacial bonding with polymer matrices remains a challenge due its surface chemical inertia.[23,26] Individual graphene layers may be formed on metal surfaces; however, for the production of bulk graphene the methods based on graphite exfoliation are preferred. The extensive exfoliation of graphite into graphene is a topic of intense research, and quite difficult to attain. It has been achieved by chemical oxidation of graphene layers in graphite, with the advantage of inducing reactivity to this material, at the expense of the electrical conductivity. In fact the graphene oxide (GO) formed presents hydroxyl and epoxy groups on both sides of the graphene plane, and carbonyl or carboxyl distributed along the edges, as represented in Figure 3.[98]

The presence of oxygen-containing functional groups render GO a biocompatible and physiologically soluble behavior, and allow stronger interactions with many polymers,[99] leading to the development of nacre-like structures, an organization widely explored in the development of tough biomimetic nanocomposites.[100] The major drawback that comes from the oxidation of graphene is the loss of the electrical conductivity.[23] However, this property may be partially restored through a chemical reduction of GO, using highly reducing reagents such as hydrazine monohydrate.[101,102]

An overview of the main works where each of the previous fillers were used is presented in Table 1. The main effects of these on the CHI polymeric matrix are also listed*.*

**Table 1.** Overview of CHI nanocomposites with different fillers.

Filler	Aim	Nanocomposite properties	Ref.
*Layered silicates*			
MMT	Study the influence of molecular weight and deacetylation degree of CHI	Intercalated CHI between the MMT layers, independently of the CHI molecular weight	[49]
	Develop porous structured scaffolds from MMT and CHI	Thermal property improved for 80:20 weight ratio MTT:CHI, decomposition onset increasing by 25°C. Silicate well dispersed in the polymer. Interlayer spacing increase from 1.2 to 1.5 nm for MMT:CHI wt ratio 20:80	[50]
	Develop nacre-like structures	CHI/MTT films densely stacked with a well-defined layered structure. Elastic modulus increase; good interfacial stress transfer ability	[51]
*Ceramic nanoparticles*			
BG NPs	Study of CHI/BG NPs potential for periodontal regeneration	Membranes with higher modulus. Ability to promote the deposition of an apatite layer upon immersion in SBF	[103]
	Produce nanocomposite coatings of BG and BG NPs with CHI	A hydroxyapatite (HA) layer was formed on all coatings; BG NPs showed higher ability to form HA	[104]
HA NPs	Study the effect of micro and nano HA for bone graft substitutes	Independently of the HA size cortical bone formation was achieved, reaching higher yield for HA NPs	[105]
	Study the mechanical properties of CHI/ HA NPs cross-linked with genipin	Tensile strength increase ~100% for HA NPs concentration up to 10 wt%, decreasing at higher HA NPs wt%	[106]
*Metallic nanoparticles*			
Ag NPs	Study the antibacterial activity of CHI/Ag NPs	Both components act synergistically against two strains of Gram-positive *Staphylococcus aureus*	[63]
Au NPs	Investigate the use of CHI matrix for glucose biosensing applications	Film characteristics were tuned by control of CHI and Ag NPs deposition conditions, achieving a biosensor with detection limit near 13 μM	[64]
ZnO NPs	Characterize the CHI/ ZnO NPs nanocomposites membranes	Achieved antibacterial activity against *Bacillus subtilis*, *E. coli* and Gram-positive bacterium *S. aureus* at 6–10 wt% ZnO NPs. Tensile strength increase (~24% for at 6 wt% ZnO NPs)	[65]
*Carbon materials*			
CNTs	Develop CHI/aligned MWNTs for neural tissue regeneration	Increase of 35.7% in Young’s modulus. Electrical conductivity increased by $10^{5}$ S m^–1^	[33]
	Coating of CHI on the CNTs surface	Increase of tensile strength for 50:50 wt% CHI/CNTs, decrease of surface electrical resistivity to 16 Ω sq^–1^	[107]
GO	Study the influence of GO on the thermal stability and mechanical properties of CHI films	Storage modulus maximized at 0.5 wt% GO and a nacre-like structure was obtained. T_g_ increased from 186.6°C to 192.5°C	[36]
	Study the mechanical behavior of CHI/graphene films	0.1–0.3 wt% of GO increased the elastic modulus over 200%	[108]
	Study the dye adsorption properties of GO/CHI composite fibers	The adsorption capacity of fuchsin acid dye onto the fibers dependent on pH	[37]
	Develop GO genipin cross-linked CHI films	Mechanical reinforcement (tensile strength); increase of resistance to enzymatic degradation; addition of GO reported to be non-toxic	[109]
	Study mechanical behavior of CHI/GO nanocomposite films in the wet state	The presence of an aqueous medium increased the tensile strength (three times higher compared to dry state)	[38]

Abbreviation: SBF, simulated body fluid.

## 3. Materials processing strategies for chitosan nanocomposites

The main techniques that have been used to produce CHI nanocomposites, namely solvent casting, freeze drying, layer-by-layer, and electrospinning are described in this section.

### 3.1. Solvent casting

**Figure 4.**
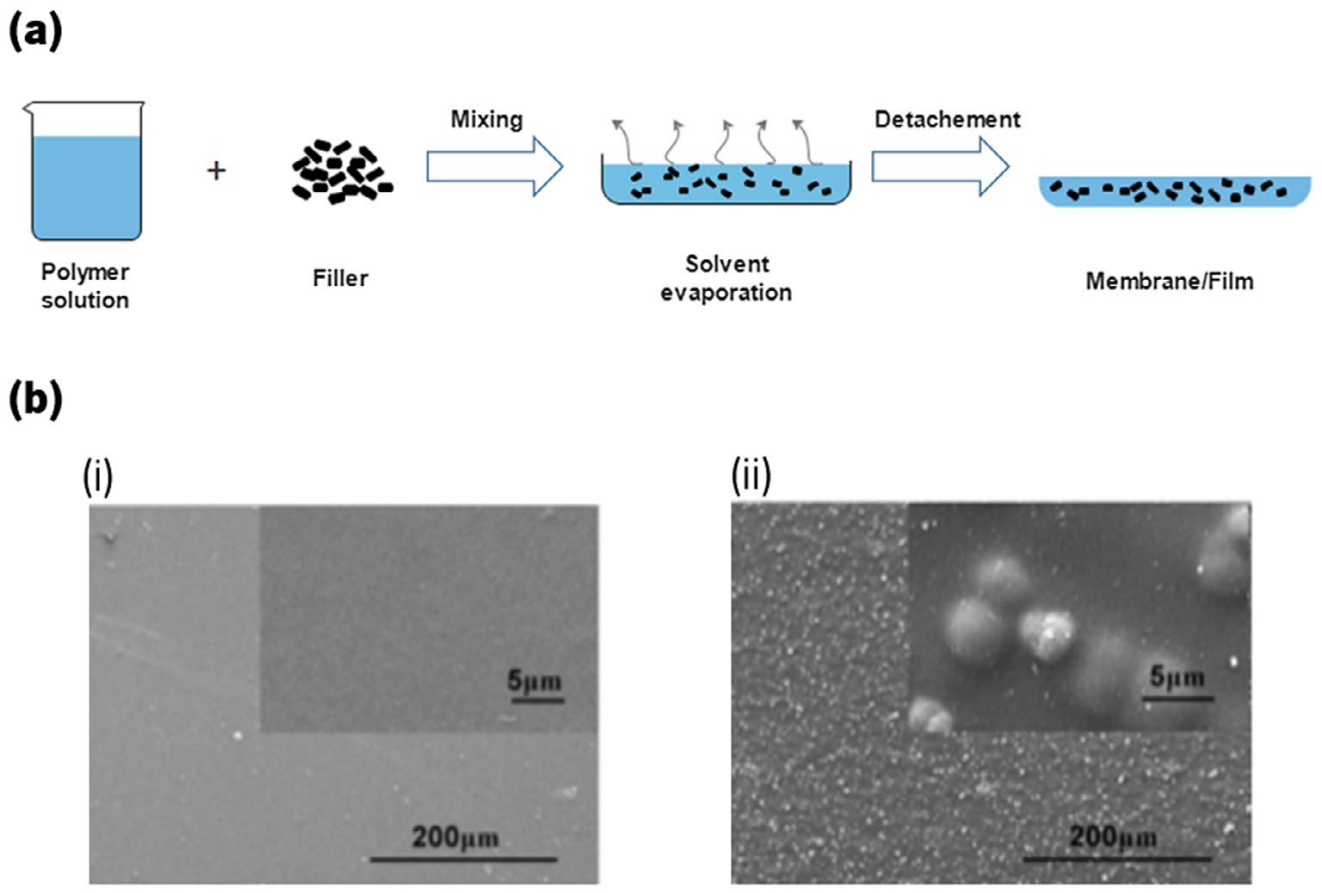
(a) Schematic representation of the procedure to obtain a membrane/film using the solvent casting method. (b) Comparison between the surface of a (i) CHI membrane and (ii) CHI/bioactive glass membrane.[115]

Solvent casting is one of the most common techniques for preparation of CHI nanocomposites films and membranes.[53,110] Typically, the polymer is dissolved in a solvent and then cast onto a surface, such as glass Petri dishes. The solvent is subsequently allowed to evaporate at room temperature or in air oven, and after that, the films/membranes are detached from the cast form (Figure 4(a)).[111]

This method is a simple approach to fabricate CHI nanocomposites, with the possibility to incorporate drugs or chemicals within the structures, with no need for expensive manufacturing equipment and high processing temperatures.[112,113] However, it still exhibits drawbacks, namely: the possible retention of residual solvents, which may cause toxicity effects, becoming harmful to transplanted cells or host tissues [114]; only simple shapes can be formed; and usually the structures present very low pore interconnectivity, unsuitable for some tissue engineering applications.[112]

There are several studies concerning nanocomposites of CHI obtained by solvent casting.[115–117] Kithva et al*.* [116] studied the formaldehyde influence on CHI–HA nanostructured composite films, including in their mechanical properties. These authors have shown that the formaldehyde presence strongly affects the interaction between CHI and HA, leading to a significant increase of the Young’s modulus and ultimate tensile strength as high as 17.3 GPa and 222 MPa, respectively, for films containing 66 wt% HA.[116] The bioactivity and viscoelasticity of CHI/Bioglass® solvent casting membranes was investigated by Caridade et al. [115] (Figure 4(b)) [116]. The data showed that after one day of immersion in SBF, apatite structures had already been developed which completely covered the samples after five days and exhibited typical cauliflower structures after seven days. The study of the viscoelasticity of the membranes by dynamic mechanical analysis revealed that storage modulus (E′) presented a continuous decrease which tended to approach to the E′ values of the pure CHI membranes. This loss of stiffness was an indication that the BG particles were being dissolved from the membranes.[115] He and co-workers,[117] exploring the same production methodology, fabricated reduced graphene oxide (rGO)/CHI composite films cross-linked with genipin.[118] They found that the addition of rGO increased the tensile strength of the films up to 72.5 MPa and also increased the thermal stability of those for an amount of 0.7%.[117]

### 3.2. Freeze drying

This method is one of the most widely used for the preparation of highly porous scaffolds by inducing thermal phase separation. Commonly, the solution temperature is lowered until solid–liquid demixing occurs, forming two different phases: frozen solvent and polymer phase. Then, the frozen solvent, through sublimation, leaves the polymeric structure forming a pore. The resultant structure can be controlled by varying the type of polymer and its concentration.[118] Anisha et al. [119] used a freeze drying process to develop antimicrobial sponges composed of CHI, hyaluronic acid and Ag NPs as a wound dressing with drug resistant bacteria.[120] The homogenous mixing of CHI, hyaluronic acid and Ag NPs followed by freeze drying resulted into a flexible and porous structure. This kind of structure presents a swelling behavior ideal for wound dressing applications, it is biodegradable and has hemostatic potential.[119] Mohandes and Salavati-Niasari,[120] using the same processing methodology, were able to synthetize a composite of CHI, GO and HA NPs, and studied its bioactivity, revealing a higher Ca and P ions release, after 14 days, than HA by itself. CHI-gelatin/MMT-HA scaffolds were prepared through freeze drying showing a nanoscale architecture with well-interconnected pores, with a mean pore size of 250 μm, similar to natural bone.[121] Sun and coworkers [122] synthetized multilayered CHI/CNTs nanocomposite films, through freeze drying and solvent casting, and found that the tensile stress and ductility of solvent casted films are higher than by freeze drying, probably due to the porosity.[123]

### 3.3. Layer-by-layer (LbL)

**Figure 5.**
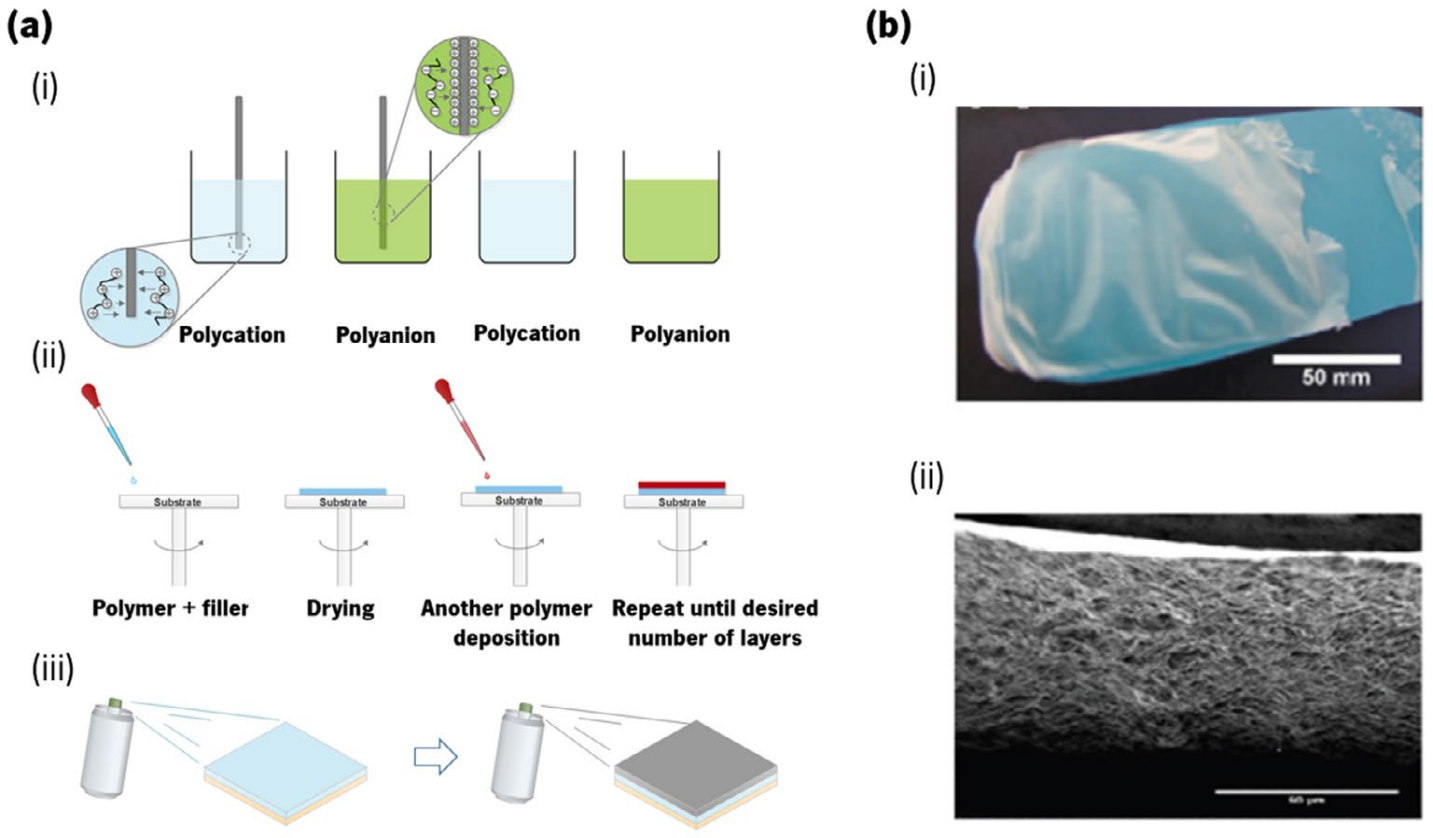
Representation of (a) three main LbL methods: (i) dip coating; (ii) spin coating and (iii) spray coating; and (b) image of a chitosan/alginate free-standing membrane, where (i) represents the membrane obtained by dip coating a polypropylene substrate after 100 cycles and (ii) its respective cross-section scanning electron microscopy (SEM) picture.[129]

Layer-by-layer (LbL) assembly, proposed by Iler in 1996 [123] and popularized by Decher,[124] is a method capable of modifying surfaces and fabricating highly ordered polymeric films and nanocomposites over different types of substrates.[125] This simple, reproducible and flexible method is based on the sequential adsorption of different macromolecular components, which are attracted to each other due to electrostatic interactions, hydrogen bonding, van der Waals forces, and electron exchange, among others.[125,126] Different LbL approaches can be used to build up a multilayer film, including dip coating, spin coating and spraying coating (Figure 5(a)).[127] Figure 5(b) shows a free-standing multilayered membrane obtained by dip coating, where it is possible to visualize a robust and layered membrane.[128]

**Figure 6.**
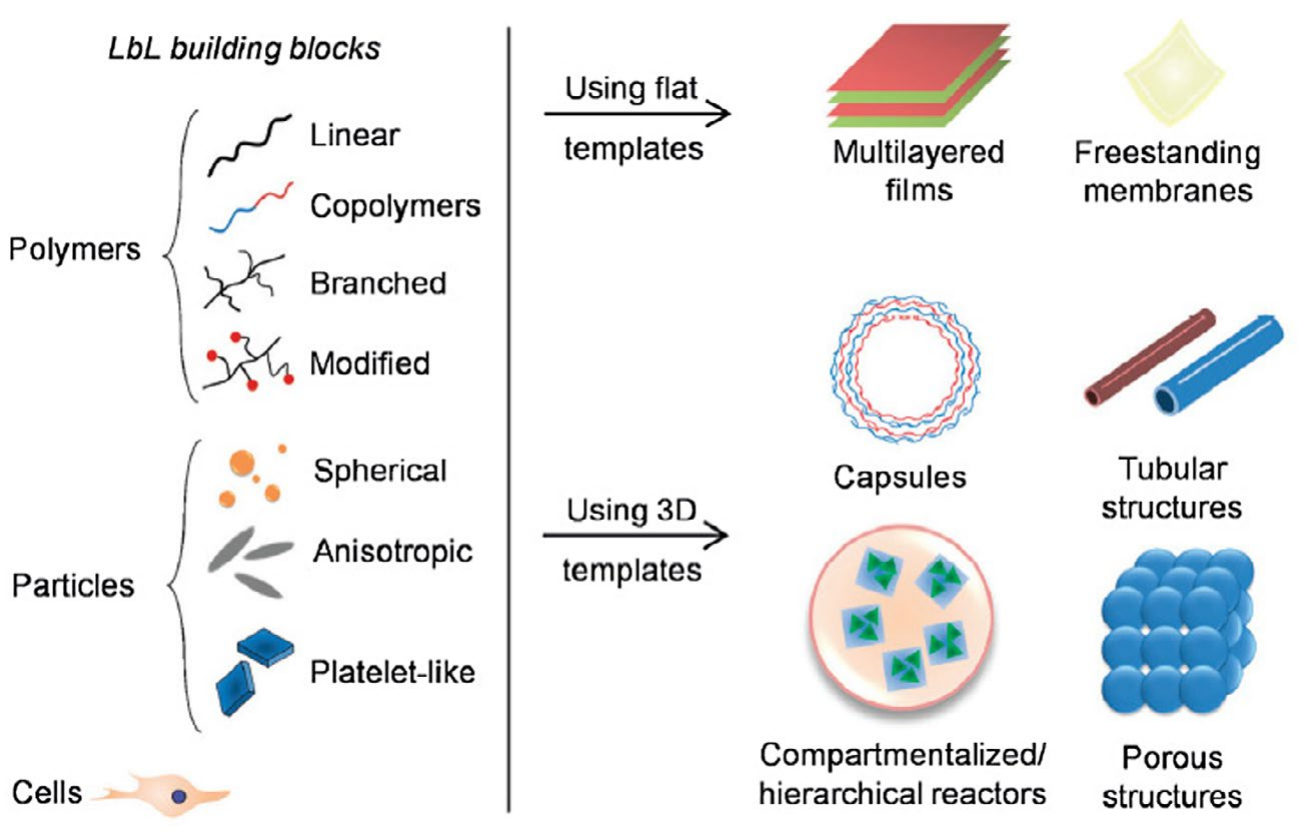
Schematic illustration of the different structures resulted from the different building blocks and substrates used in the LbL process.[130]

Due to its versatility and great availability of building blocks (e.g. CNTs, clays, NPs, polymers), this technology allows fabrication of multilayered devices of any nature, size, shape, and chemical composition, assuring the development of nanostructures with desired geometries and functionalities (Figure 6).[129] In addition, the properties of multilayered devices can be tuned through solution pH, temperature, or ionic strength.[126]

Yang et al. [130] used the LbL assembly approach to fabricate BG NPs/CHI/alginate (ALG) scaffolds.[131] The bioactive glass scaffolds were immersed in each biopolymer and the results showed that as the CHI and ALG were infiltrated in the porous scaffold, a decline in the porosity was achieved, reaching 1.8% less at the end of three cycles. This continuous assembly between the BG scaffold and both biopolymers also resulted in an increase of the mechanical properties, achieving an elastic modulus of 80 MPa and a compressive modulus four times higher than the BG scaffolds.[130] Pavinatto and coworkers [131] assembled CHI/MWNTs films, built on a fluorine doped tin oxide electrode, to study the detection of 17α-ethinylestrol.[132] They found a faster electron transfer kinetics and good detection limit of 0.09 µmol l^–1^.[131] Couto et al*.* [132] modified LbL coatings using CHI and BG NPs in order to mimic the organic-inorganic structure of nacre.[133] These authors using quartz crystal microbalance showed that this methodology may be used to produce tunable and viscoelastic nanostructured multilayers upon increasing the number of LbL cycles. Moreover, it was shown that after 14 days of immersion in SBF, apatite-like layers distributed over the entire composite surface were achieved exhibiting cauliflower morphology. X-ray diffraction (XRD) measurements also confirmed crystalline structure of the calcium phosphate layer.[132]

### 3.4. Electrospinning

**Figure 7.**
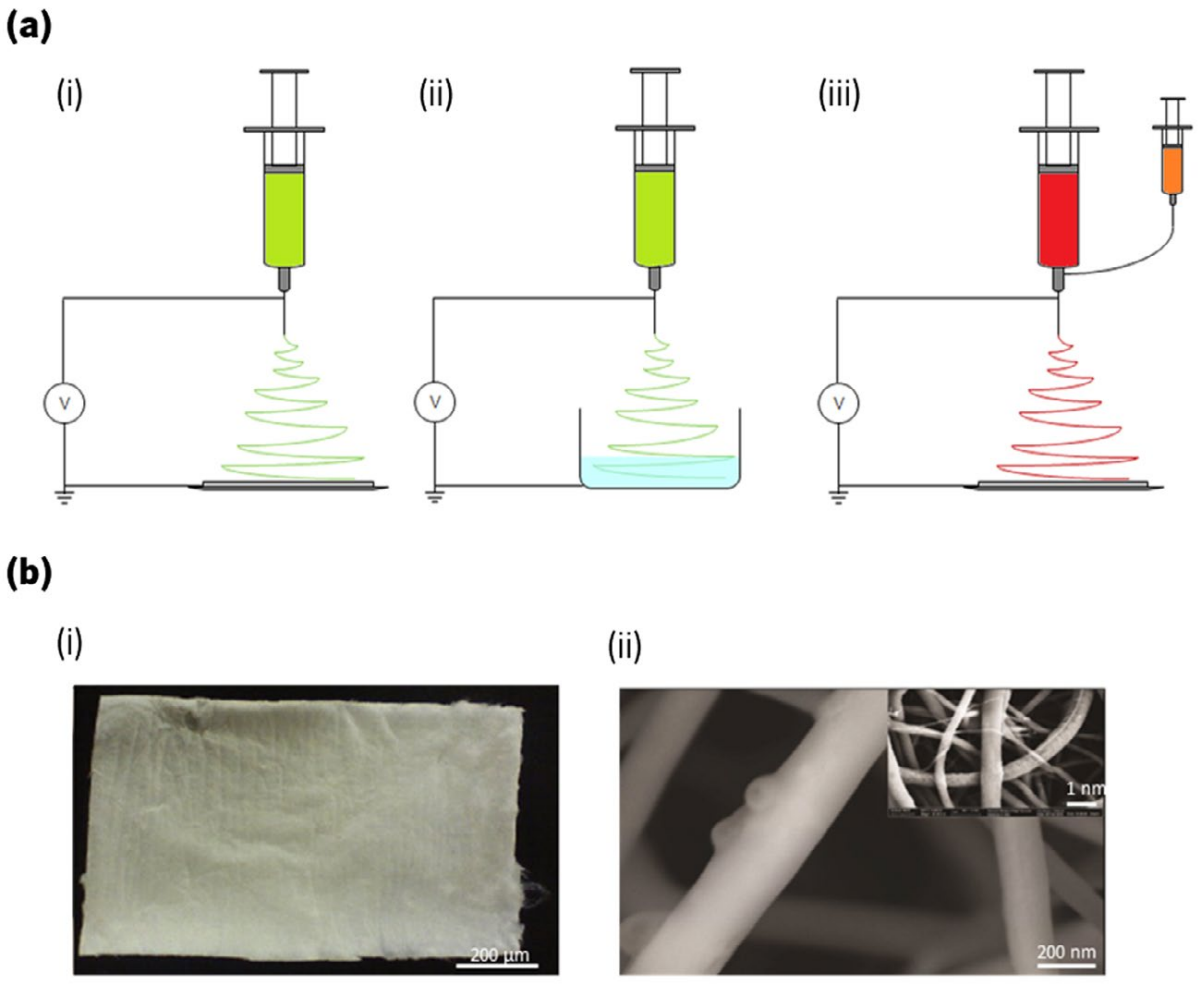
(a) Representation of the different electrospinning approaches, namely (i) wet–dry spinning, (ii) wet–wet spinning and (iii) co-axial electrospinning. (b) Representation of a (i) macroscopic electrospun chitosan fiber mat and (ii) chitosan/hydroxyapatite nanoparticles fibers morphology obtained by SEM with respective insert image at lower magnification.[136]

Electrospinning represents a suitable technique to produce fibers with diameters in the nm–µm length scale, since it allows morphology, porosity and composition to be controlled using relatively unsophisticated equipment. Typically, the electrospinning process uses an electric field created between the polymer solution and the collector, which generates internal repulsive forces in the polymer solution and, at a critical point, causes the expulsion of the polymer solution in shape of fibers towards the collector.[133,134] There are three different types of electrospinning: wet–dry electrospinning, wet–wet electrospinning and co-axial electrospinning (Figure 7(a)).[110] The major difference between the first two methods is that the wet–dry method uses a volatile solvent which evaporates as the fibers are spun through the collector, while the wet–wet method spins a non-volatile solvent to a collector with a second solvent. Regarding the last method, it is possible to obtain fibers with a core-sheath structure, as two different components can be spun at the same time.[110]

This cost-effective and facile technique has been widely studied for nanocomposite production for wound dressings, medical implants and scaffolds (Figure 7(b)).[111,135,136] For the particular case of soluble polysaccharides such as CHI, the wet–dry methodology is the most used. Bai et al. [137] explored the development of electrospun CHI nanofibers functionalized with a quaternary amine, N-[(2-hydroxy-3-trimethylammonium) propyl] chitosan chloride (HTCC), to adsorb and reduce virus presence. Because of the difficulties in electrospinning HTCC alone, these authors used graphene as additive. They found that the good interaction between the graphene and the highly charged trimethyl amine group on the HTCC allowed a 95% reduction of porcine parvovirus due to the higher amount of HTCC that was able to reach the collector.[137] Najafabadi et al. [138] studied the ability of CHI and GO to absorb metal ions such as ${\text{Cu}}^{2+}$, ${\text{Pb}}^{2+}$, and ${\text{Cr}}^{2+}$, showing that the obtained nanocomposites were able to absorb such ions, but with a decreasing capacity when pH values were above 6. Moreover, the adsorbent could be used up to five times without losing its initial adsorption capacity.[138] Another example of electrospinning using for nanocomposites development, comes from the work done by Lee et al. [139] using Ag NPs together with CHI; these authors were able to obtain homogenous nanofibers structures with a high efficiency against bacterial growth, particularly for Ag NPs amount of 2 wt%.[140] Mahdieh and coworkers [140] electrospun CHI and MWNTs, obtaining nanofiber nanocomposites with a conductivity of 9 × 10^−5^ S cm_-1_, showing great potential for biomedical applications.

## 4. Biomedical applications

**Figure 8.**
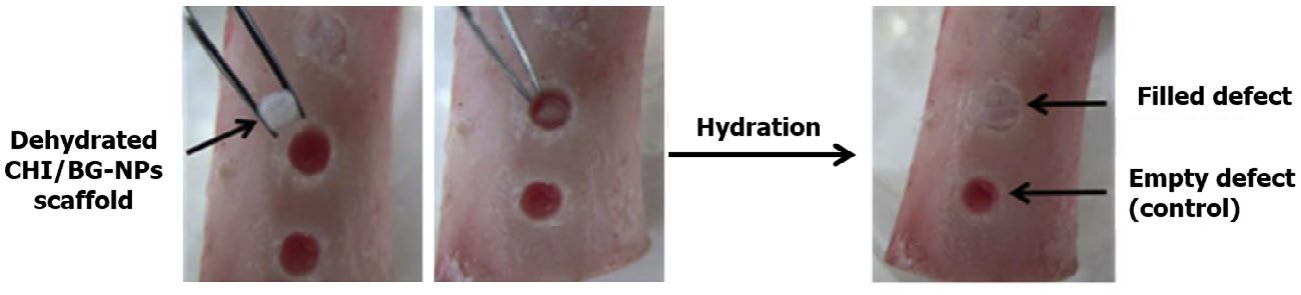
Representation of a possible application of CHI nanocomposite for bone regeneration. CHI/BG-NPs scaffolds were used to fill the pig femur bone defect when this was hydrated.[148]

The composition, architecture, and mechanical properties of nanocomposites are important to engineer implants that can balance the degradation with remodeling of regenerated tissue.[52,141] In fact, the development of biomimetic nanocomposites with different inorganic nanofillers, incorporated within polymeric materials, has been extensively studied.[142,143] Natural and synthetic polymeric nanocomposites represent powerful technologies for biomedical applications, namely for tissue regeneration and controlled drug delivery.[144,145] Several materials have been found to be suitable for biomimetic nanocomposites (Figure 8).[17,146]

Herein, the major biomedical applications where CHI nanocomposites can be applied are described, as well as a brief overview of the main works done under this scope in the last five years. Table 2 gives a brief overview of the works concerning CHI nanocomposites for biomedical applications, some of which are analyzed next.

**Table 2.** Chitosan based nanocomposites for different biomedical applications.

Application	Composition	Processing technique	Shape	Ref.
Biosensing	CHI/DNA/MWNTs	Spin coating	Film	[149]
	CHI/GO bond to 5’amine single strand DNA	Spin coating	Film	[150]
	CHI/Au NPs/hemoglobin	Dip coating	Membrane	[151]
	CHI/Au NPs linked to cytochrome c and glucose oxidase	Electrodeposition	Film	[152]
Bone regeneration	CHI/HA NPs loaded with icariin	Solvent casting	3D scaffold	[153]
	CHI/HA NPs/ collagen	Freeze drying	3D scaffold	[154]
	CHI/HA NPs/SWNTs	Freeze drying	3D scaffold	[155]
	CHI/BG NPs	Freeze drying	3D scaffold	[148]
	CHI/GO	Freeze drying	3D scaffold	[156]
Drug delivery	CHI/layered silicate loaded with doxorubicin	Freeze drying	3D scaffold	[157]
	ALG/CHI/ HA NPs loaded with doxorubicin	Solvent casting	Hydrogel	[158]
	CHI/GO loaded with fluorescein sodium	Solvent casting	Membrane	[159]
	CHI/MMT loaded with oxytetracycline	Freeze drying	Scaffold	[160]
Tissue engineering	CHI/CNTs	Freeze drying	3D scaffold	[161]
	CHI/graphene	Solvent casting	Hydrogels	[162]
	CHI/HA NPs	Electrospinning	Membrane	[163,164]
	CHI/CNTs	Solvent casting	Hydrogel	[165]
	CHI/carbon nanofibers	Freeze drying	3D scaffold	[166]
	CHI/TiO_2_ NPs	Solvent casting	Membrane	[167]
Wound healing	CHI/poly(ethylene glycol)/ZnO/Ag NPs	Solvent casting	Film	[168]
	CHI/PVA/graphene	Electrospinning	Membrane	[169]
	CHI/MMT loaded with silver sulfadiazine	Solvent casting	Membrane	[170]
	CHI/Ag NPs	Solvent casting	Membrane	[171]
	CHI/Ag-MMT	Solvent casting	Film	[172]
	CHI/reduced GO	Solvent casting	Film	[173]

Abbreviation: PVA, poly(vinyl alcohol).

### 4.1. Bone tissue engineering

Bone is a mineralized connective tissue formed by osteoblasts.[173] The fundamental subunit of bone structure is mineralized collagen fibril that consists of self-assembled triple helices of collagen, carbonated apatite and water molecules.[174,175] Different types of bone grafts can be used for fracture damage, e.g. autografts, allografts and synthetic bone grafts.[176] Autografting is considered the gold standard for bone repair, however it exhibits significant disadvantages such as limited availability, donor site morbidity and risk of disease transfer from donor to recipient.[177] Allografts show some risks, e.g. lack of osteoinduction and osteoconduction, risk of disease transmission and insufficient mechanical properties. Thus the development of synthetic bone grafts where these drawbacks are overcome represents a tremendous need for bone repair,[178] in particular through the use of biomimetic devices exhibiting osteoconductive properties.[174]

Im et al. [154] developed a 3D porous CHI scaffold, using a freeze drying technique, with different amounts of nanosized HA (nHA) and SWNTs, to induce the growth of human osteoblasts. When compared with conventional CHI scaffolds, the introduction of nHA (20 wt%) and SWNTs (1 wt%) allowed a significant enhancement of the tensile and compressive moduli. Cellular assays performed to test the cell adhesion demonstrated that the introduction of nHA plays an important role in scaffold cytocompatibility, as the osteoblast adhesion increased by 59%. Moreover, when SWNTs were added, a great improvement in cell adhesion was observed. This increase suggests that the addition of SWNTs to nHA/CHI scaffold may have a synergistic effect, improving their cytocompatibility properties.[154] CHI has been combined with BG nanoparticles to produce scaffolds with hydration induced shape memory and biomineralization ability.[147] The developed scaffolds had an osteoconductive character, and SEM images revealed an apatite layer and cauliflower like-structures, with almost total surface coverage after seven days. Concerning the shape memory ability, the nanocomposite scaffolds had better shape memory than pure CHI, showing a shape recovery of 89.9%, after a 30% strain was applied.[147] The potential use of electrospun nHA/CHI fibers seeded with bone marrow mesenchymal stem cells (BMSCs) for bone regeneration was studied [163]; after seven days it was found that BMSCs exhibited a spindle morphology on nHA/CHI, but a spherical shape on CHI, revealing an important effect of HA for supporting cells spreading. Polymerase chain reaction test of mRNA for integrin subunits were notably up-regulated with cells cultured on nHA/CHI compared to CHI after 14 days, highlighting the great potential of using the HA/CHI nanocomposite nanofibers for bone tissue engineering applications.[163] Depan and coworkers [155] investigated the biological response of osteoblasts cells on CHI/GO scaffolds as well as the mechanical properties of the produced nanocomposite material. According to this work, an increase of 44% in the elastic modulus was verified and an enhancement in the cell attachment, proliferation and growth was achieved.[155]

### 4.2. Drug delivery

The increasing limitation of standard drugs caused by nonspecific cell targeting and tissue biodistribution, allied with their rapid metabolism and excretion, resulted in a great demand for systems with optimized performance.[179,180] Nanocarriers have emerged as one of the most potential drug delivery devices as they are able to interact with the cell membranes and enter into the cells by endocytosis, escaping to the endosomal compartment, releasing the drug in cytosolic compartments.[181] The main requisites to build these systems are biodegradability and biocompatibility, which typically are provided by natural polymers such as CHI.[182,183]

The potential application of GO/CHI nanocomposites, obtained by solvent casting, for drug delivery systems, namely for transdermal drug delivery has also been focus of study.[184] Different amounts of GO that should be used to provide both a good mechanical performance and a good release profile of the drug were pointed out as essential key factors. Justin and Chen [184] found that an amount of 2 wt% GO showed the best conditions, allowing an increasing in the Young’s modulus from 1 to 1.3 GPa, and at the same time an enhancement in the tensile strength and elongation at break. Concerning the drug release (fluorescein sodium), it was shown that this phenomenon was dependent on the loading ratio of the drug to GO and from the pH of the medium. These authors found that the optimal loading ratio was 45.6%, allowing a 72% releasing increase in a shorter time than CHI.[184] When tested in acidic conditions the release decreased, showing the great potential of these nanocomposites for a selective release.[184] Furthermore, Salcedo et al. [159] explored the ability of CHI nanocomposites systems with MMT to delivery oxytetracycline via oral route. The *in vitro* and oxytetracycline absorption was explored with CaCo-2 cell line and it was shown that the concentrations of nanocomposites with higher average values of cell viability was achieved for a CHI/MMT of 0.25 mg ml^–1^ and for oxytetracycline 0.0375 mg ml^–1^ amount. Confocal laser scanning microscopy revealed that the nanocomposite particles are into the depth of the cell substrate, in contact with cell nuclei, indicating an actual internalization and not only a membrane interaction or a deposition of the particles on cell substrate.[159] Recently, Mo et al*.* [185] combined doxurobicin (DOX), an anticancer drug, with hyaluronan/CHI/SWNTs. They explored the use of SWNTs as vehicles for DOX, CHI to enhance water solubility and pH sensibility and in particular hyaluronan was used as it constitutes a ligand of CD44 receptor, which are overexpressed in cancer cells. The SWNT/CHI/hyaluronan/DOX presented lower toxicity to fibroblasts (representative of normal cells) than for HeLa cells (cancer cells representatives). The DOX release showed a high rate when exposed to a pH 5.5 reaching a total release of 85%, when compared with the medium at pH 7.4, showing a selective delivery system mechanism.[185]

### 4.3. Soft tissue applications

CHI nanocomposites have also been investigated for soft tissue applications. Wound healing properties of nanocomposites are among the most researched properties for biomedical applications. Wound healing is a dynamic process consisting of four overlapping, and programmed phases, namely: homoeostasis, inflammation, proliferation and tissue remodeling.[186,187] The microenvironment of the wound healing process is complex and involves the interaction of a large number of different types of cells and molecules.[188] To achieve and promote a better healing process, wound dressings play an important role and should include some essential features, such as: biocompatibility; the ability to prevent bleeding and dehydration of the wound; the ability to keep a favorable moist environment; protection of the wound against external contamination; permeability to gas and fluid exchanges; the ability to absorb exudates from the wound area; thermal isolation; non-toxicity; and a non-allergenic profile.[189,190] Hydrogels based on pure CHI with high toughness may be obtained using, for example, double-network strategies [191]; however, the use of nanocomposites based on CHI matrices are much more usual. Considering these features, Lu et al. [168] reported the use of CHI-PVA/graphene nanofibers, produced by electrospinning, for wound healing applications. These authors tested the potential of these membranes on mouse and rabbit skin wounds and found that after five days, the wound area significantly decreased and at the end of 10 days the skin was completely recovered, while for membranes without graphene, these wound areas still exist.[168] Aguzzi and coworkers [169] explored the use of CHI/MMT nanocomposites loaded with silver sulfadiazine for the same purposes. They demonstrated a successful loading of the silver sulfazadine in the nanocomposite structure, as XRD tests have shown no free drug in the composite matrix, revealing that the intercalated nanocomposite was formed by insertion of drug and/or polymer molecules, with a homogeneous dispersion in the nanocomposite structures.[169]

Associated with the wound healing process, there is an important property of materials that can also accelerate and benefit the recovery of a tissue, i.e. antimicrobial properties. Usually, microbes are able to multiply faster and hinder wound healing, and thus the inhibition of these microorganisms results in better wound dressing materials.[168] Gonzalez-Campos et al. [170] produced CHI/Ag NPs films with antimicrobial activity, using solvent casting, and studied their effect on Gram-positive and Gram-negative bacteria. It was found that the highest antibacterial activity occurred for films with 3 wt% of Ag NPs, and above this concentration, the bactericide effect tends to decrease. Moreover, these authors found that the presence of Ag NPs in the CHI polymeric matrix led to appearance of silver ions on the nanocomposite, giving a conductive character to CHI films.[170] Lavorgna et al. [171] prepared nanocomposites using a CHI matrix with silver-montmorillonite antimicrobial behavior by replacing ${\text{Na}}^{+}$ ions of natural MMT with silver ions. They were able to achieve enhanced mechanical performance, but more importantly they have shown that after 24 h a significant delay in *Pseudomonas aeruginosa* was obtained.[171] Lim et al. [172] also explored the ability of rGO and CHI nanocomposites to retard the growth of *Pseudomonas aeruginosa*. The achieved data revealed that bacterial growth was not dependent on the concentration and size of rGO and could be completely suppressed by the low concentration of rGO in the chitosan solution, leading to a maximum viability loss of 100%.[172]

### 4.4. Biosensing

The detection of biologically active molecules is extremely important for biomedical purposes.[90] Nanomaterials have demonstrated great ability for biosensing applications as they are able to achieve enhanced performances with increased sensitivities and lowered detection limits of several orders of magnitudes.[192] The production of bioelectrodes is often dependent of the large surface-to-volume ratio and good electrochemical activity. As shown by Singh and co-workers,[149] CHI/GO nanocomposites have demonstrated the ability to detect DNA for rapid and sensitive detection of typhoid, using a *Salmonella typhi* specific 5′-amine labeled single strand (ss) DNA (5′NH_2_-ssDNA), covalently bound through CHI/GO by glutaraldehyde*.* The produced bioelectrode demonstrated to distinguish complementary and non-complementary sequences, which in part may be related with the essential characteristics previously mentioned but also due to good biocompatibility of CHI, which enhances the DNA immobilization and facilitates electron transfer between DNA and electrode surface.[149] A glucose biosensor developed with cytochrome c and glucose oxidase entrapped on Au NPs and CHI and constructed on a glassy carbon electrode was fabricated by Song et al*.* [151]. They demonstrated that the deposition of CHI/Au NPs led to an increase of the roughness to 9.5 ± 0.1 nm, which revealed to be important to provide a large surface-to-volume ratio. In addition, a higher sensitivity to glucose and a lower detection limit was obtained.[151] Zhang et al. [150] reported the fabrication of a hemoglobin/Au NPs/CHI/graphene biosensor developed on a glassy carbon electrode for hydrogen peroxide detection. The electron transfer properties of the biosensor were analyzed using electrochemical impedance spectroscopy. The data showed that the use of hemoglobin, Au NPs and graphene improve the electron transfer, reducing the transfer resistance provide by CHI. In addition, a low detection limit (0.35 µM), a good stability (94%) for over one month and a high sensitivity (347.1 $\text{mA}/{\text{cm}}^{2}M$) was found for these biosensors.[150] More efforts should be put in combining sensing ability in therapeutic strategies, in order to develop nanocomposites processed as NPs, fibers or coatings for theragnosis applications.

## 5. Conclusions

CHI, a promising biomaterial that by itself presents outstanding properties, revealed to improve the nanotechnology field when reinforced with various other nano-sized fillers. Depending on the application, the characteristics of each CHI nanocomposites can be controlled, designed and modulated regarding the target tissue.

Most nanocomposites result from the need to improve a critical feature that plays a major role in some specific tissue, such as the mechanical properties for bone regeneration, antibacterial activity for wound healing or improved drug delivery for a targeted treatment. Independently of the target, the nanocomposite performance is closely related with the good dispersion of fillers within its polymeric matrix. The successful dispersion allows a good polymer/filler interface that, independently of the application, always results in high specific interfacial area. Thus, an optimal CHI/filler interaction is the required key to benefit from nanocomposites with full potential.

Successful findings have been reported such as osteogenic and osteoconductive properties when BG NPs are used. In the presence of fillers like CNTs or graphene, enhanced mechanical, thermal and conductive properties are achieved and with incorporation of Ag NPs antibacterial effects were also observed. However, although significant progress has been made in understanding these nanocomposites, with promising results being demonstrated by the described works, a lack of information exists concerning several aspects. As described in this review, morphological aspects are very important for the improvement of the nanocomposites, with the optimal properties depending on the application. Thus, features such as dispersion of the nanofiller within the CHI matrix should be the focus of more extensive studies. In fact, new strategies such as the CNT alignment before nanocomposites fabrication is being explored. Moreover, it would be interesting to pursue new studies concerning other material features such as the degradation of the nanocomposites. Such improvements could be extremely important, once the durability and toxicity of the device are improved. Other important aspects that require further development concern *in vivo* or pre-clinical studies. Thus, these nanocomposite devices have a promising future, but significant and important steps should be taken to understand the *in vivo* interaction of these nanocomposite devices with the host tissue. At the same time some key points should be further explored such as the ability of these materials to be sterilized using conventional methods, for application in daily clinical practice. Along with the aforementioned aspects, insights into unresolved issues such as the emergence of the analytical protocols for quality assessment of CHI should be addressed both for scientific and market purposes.

The vast opportunities shown by these materials, allied with their incredible nanotechnology potential, is expected to revolutionize the biomedical field in the near future.

## Disclosure statement

No potential conflict of interest was reported by the authors.
